# Childhood trauma, specifically emotional abuse, is associated with psychiatric symptom severity in drug‑naïve first‑episode patients with schizophrenia

**DOI:** 10.1038/s41598-026-63324-2

**Published:** 2026-07-23

**Authors:** Shilong Jia, Hailuo Liu, Renyun Zhang, Jie Liu, Sufang Qi, Mengmeng Sun, Limin Yang

**Affiliations:** 1 Department of Psychiatry, Shandong Mental Health Center, Shandong University, Jinan, Shandong China; 2 Department of Pediatrics, Jinan Lixia District People’s Hospital, Jinan, Shandong China

**Keywords:** Adverse childhood experiences, Psychiatric symptoms, Emotional abuse, Diseases, Health care, Medical research, Psychology, Psychology

## Abstract

Schizophrenia (SCZ) is a highly heterogeneous disorder. Childhood trauma (CT) is a well‑established risk factor, although the contribution of specific CT dimensions remains incompletely understood. This study systematically investigated which CT dimensions were most strongly associated with symptom severity in drug‑naïve first‑episode SCZ patients. In a cross‑sectional design, 284 patients were classified into a SCZ‑ct group (*n* = 234) and a SCZ‑nct group (*n* = 50) based on Childhood Trauma Questionnaire. Symptom severity was assessed using the Positive and Negative Syndrome Scale. Hierarchical regression analyses adjusted for demographic and clinical covariates were performed, with rater effects examined via a linear mixed‑effects model. The SCZ‑ct group exhibited significantly higher total and positive symptom scores, whereas negative symptom scores did not differ. Higher total CTQ scores were associated with greater overall symptom severity. Emotional abuse was independently associated with total PANSS scores after adjustment for other CT dimensions and covariates, and this association remained robust after additionally accounting for rater effects. These findings support the potential value of addressing childhood trauma, particularly emotional abuse, in both clinical practice and public health strategies.

**Supplementary Information:**

The online version contains supplementary material available at 10.1038/s41598-026-63324-2.

## Introduction

Schizophrenia (SCZ) is a severe and chronic psychiatric disorder affecting approximately 0.28% of the global population, associated with substantial disability, reduced life expectancy and marked social and economic burden^1^. Clinically, SCZ is defined by a complex and variable symptom profile, encompassing positive symptoms (e.g., hallucinations and delusions), negative symptoms (e.g., blunted affect and avolition) and general psychopathology (e.g., anxiety and cognitive disturbances)^2,3^.

At present, the clinical diagnosis and assessment of SCZ rely primarily on symptom-based evaluation, which poses major challenges for accurate diagnosis, treatment selection and prognosis prediction. As a result, a substantial proportion of patients continue to experience persistent symptoms, suboptimal treatment response, or limited functional recovery^4–6^.

In addition to well-established biological determinants such as genetic vulnerability and neurobiological abnormalities, accumulating evidence highlights the importance of psychosocial factors in shaping symptom severity and clinical trajectories in SCZ^7^. Identifying modifiable psychosocial factors that influence symptom expression may provide novel opportunities to improve treatment outcomes and guide individualized intervention approaches.

One potential psychosocial factor that may contribute to symptom severity among patients with SCZ is childhood trauma (CT). CT refers to adverse experiences occurring before the age of 16, including emotional, physical and sexual abuse, as well as emotional and physical neglect^8,9^. Extensive epidemiological research has demonstrated that individuals exposed to CT have a significantly increased risk of developing SCZ compared with those without such exposure^10^. Beyond its role in disease onset, CT has also been associated with greater overall psychiatric symptom severity in adult patients with established SCZ, including more severe psychotic symptoms and poorer functional outcomes^11^.

Despite growing interest in this topic, important gaps remain in the literature regarding the relationship between CT and symptom severity in SCZ. First, although cumulative CT exposure has been repeatedly linked to worse clinical outcomes, many studies aggregate different CT dimensions into a single composite score or focus on a single trauma subtype, thereby limiting the identification of the CT dimension most strongly associated with symptom severity^12^. Second, findings regarding which symptom domains are most affected by CT are inconsistent. While some studies report a predominant association with positive symptoms, others report associations with negative symptoms^13,14^. Third, prior research has often been confounded by the effects of long-term medication and disease chronicity, making it difficult to isolate the specific contribution of early-life adversity to psychopathology.

Therefore, identifying whether CT is preferentially associated with specific symptom domains, and determining which CT dimension is most robustly associated with symptom severity, is essential for understanding how psychosocial adversity may relate to the clinical heterogeneity of SCZ. To address these gaps, the present study focused on a cohort of patients experiencing a first episode of SCZ who were naive to medication. This design minimizes the confounding effects of medication, allowing for a precise evaluation of the relationship between trauma and symptoms.

Specifically, this study aimed to address two pivotal questions: (1) Do SCZ patients with a history of CT exhibit a distinct symptom profile compared to those without? (2) Which specific CT dimension is most strongly associated with symptom severity?

## Methods

### Participants and procedures

The study population consisted of patients who hospitalized at Shandong Mental Health Center between January 2024 and November 2024. Specifically, only patients diagnosed with drug-naïve first-episode schizophrenia were included. The inclusion criteria were as follows: (1) met the diagnostic criteria for SCZ according to the DSM-5 criteria; (2) aged 18–60 years, regardless of gender; (3) had a Positive and Negative Syndrome Scale (PANSS) score ≥ 70; (4) had no prior medication history. The exclusion criteria were as follows: (1) severe physical disease or brain disease; (2) a history of psychoactive substance abuse; (3) current pregnancy or lactation; (4) mental retardation or other causes of impaired intelligence.

### Data collection

To standardize the assessment process, all assessors, who were qualified psychiatrists, completed standardized training administered by the study manager before data collection. Eligible participants provided written informed consent following a comprehensive explanation of the study. All scales were completed independently by participants. If any questions emerged during completion, assessors provided only standardized responses to avoid response bias. All data were recorded and double-checked by dedicated research personnel to ensure accuracy and completeness.

### Demographic data

Demographic data, including gender, age, duration of mental disorder, educational level and family history of mental disorder, were collected using a self-developed scale.

### Positive and negative syndrome scale

The PANSS is a standardized tool for assessing symptom severity in patients with SCZ^15^. It consists of 30 items across three subscales: the positive symptom subscale (7 items), negative symptom subscale (7 items) and general psychopathology symptom subscale (16 items). Each item is rated on a 7-point Likert scale (1 = absent to 7 = extreme) based on symptom presentation over the previous week, with total scores ranging from 30 to 210 (higher scores indicate more severe symptoms).

### Childhood trauma questionnaire

The childhood trauma questionnaire (CTQ) comprises 28 items assessing abuse and traumatic experiences prior to age 16 and is organized into five dimensions: emotional abuse, physical abuse, sexual abuse, emotional neglect and physical neglect^16^. Individuals meeting any one of the following scoring criteria are considered to have experienced CT, whereas those not meeting these criteria are considered to not have experienced CT: emotional abuse score ≥ 13, physical abuse score ≥ 10, sexual abuse score ≥ 8, emotional neglect score ≥ 15, and physical neglect score ≥ 10. Based on these criteria, 284 patients were divided into a CT group (SCZ-ct group) and a non-CT group (SCZ-nct group).

### Statistical analysis

Statistical analysis was performed using SPSS 26.0 (IBM Corp., Armonk, NY, USA). A two-tailed α = 0.05 was used as the significance level, with *p* < 0.05 considered statistically significant. Continuous variables were presented as the mean ± standard deviation (SD) and categorical variables as frequencies and percentages (n/%). Normality of continuous variables was assessed using skewness and kurtosis tests. Between SCZ-ct and SCZ-nct comparisons were performed using independent-samples t tests for continuous variables and chi-square (χ^2^) tests for categorical variables.

Hierarchical linear regression was used to examine the associations between childhood trauma and total PANSS scores. Before modelling, categorical covariates were dummy‑coded: gender (reference = female), family history of mental disorder (reference = negative), and educational level (reference = senior high school). Continuous covariates (age, duration of mental disorder) were entered directly. Three regression models were fitted sequentially. Model 1 examined the association between the total CTQ score and total PANSS score in a simple univariate regression. To identify the specific CTQ subdimension most strongly associated with symptom severity, univariate regressions were first run for each of the five CTQ subscales. Model 2 was then constructed as a two‑block hierarchical regression: the subscale showing the strongest bivariate association with total PANSS scores was entered in Block 1, and the remaining four subscales were entered simultaneously in Block 2 to test whether any other dimension explained incremental variance. All five subscales were retained regardless of their individual significance levels, so that each predictor’s contribution was evaluated within a fixed framework rather than selected on the basis of p‑values. Model 3 extended this framework by incorporating demographic and clinical covariates. Blocks 1 and 2 remained identical to Model 2, while Block 3 added age, duration of mental disorder, gender, family history of mental disorder, and educational level. Collinearity diagnostics were examined for all models; all variance inflation factors were below 1.25. Model fit was evaluated using R^2^, adjusted R^2^, and the overall F‑test. Power analyses performed with G*Power 3.1 confirmed that the sample provided adequate statistical power for the primary analyses.

To account for multiple comparisons, the false discovery rate (FDR) correction was applied to the between‑group comparisons (9 tests) and to the univariate regressions of individual CTQ dimensions on total PANSS score (5 tests). FDR‑adjusted p‑values are reported alongside unadjusted p‑values where applicable. The hierarchical regression models (Model 1–3) tested pre‑specified hypotheses within a single analytical framework and were not subject to additional multiple comparison correction.

To explore potential rater-related bias in total PANSS scores, a linear mixed-effects model was constructed, in which all five CTQ subscales and demographic and clinical covariates (age, gender, illness duration, educational level, and family history of psychiatric disorders) were included as fixed effects, while rater identity was specified as a random intercept. Additionally, the intraclass correlation coefficient (ICC) derived from the random intercept variance only quantifies the proportion of total outcome variance explained by between-rater differences, rather than serving as formal inter-rater reliability metrics. Formal inter-rater reliability assessment was not feasible in this study, as multiple raters did not independently assess identical participants. This mixed-effects model was merely a supplementary sensitivity analysis to isolate rater-attributable variance after adjusting for all fixed-effect predictors, and the ICC only reflected the magnitude of measurement variability introduced by assessor differences.

## Results

### Sociodemographic characteristics

To elucidate the specific links between childhood trauma and schizophrenia symptoms, we first sought to assemble a study cohort of adult SCZ patients who either had or had not experienced CT. A total of 284 patients with SCZ were enrolled in this study, including 234 patients in the SCZ-ct group and 50 patients in the SCZ-nct group (Table 1). Demographic and clinical characteristics of the study participants were compared between the two groups.

**Table 1:** The characteristics of respondents.

	SCZ-ct (*n* = 234)	SCZ-nct (*n* = 50)	t/X^2^	*p*
Age, years, mean (SD)	28.24 (7.90)	29.58 (8.32)	1.082	0.28
Gender, n (%)			1.56	0.21
Male	102 (43.6)	17 (34.0)		
Female	132 (56.4)	33 (66.0)		
Educational level, n (%)			4.10	0.25
Primary school	11 (4.7)	0		
Junior high school	57 (24.4)	10 (20.0)		
Senior high school	128 (54.7)	28 (56.0)		
College or above	38 (16.2)	12 (24.0)		
Family history of mental disorder, n (%)			0.16	0.69
Positive	77 (32.9)	15 (30.0)		
Negative	157 (67.1)	35 (70.0)		
Duration of mental disorder, years, mean (SD)	1.60 (3.53)	1.16 (1.32)	-0.86	0.39

Regarding gender, there were 102 males (43.6%) and 132 females (56.4%) in the SCZ-ct group, and 17 males (34.0%) and 33 females (66.0%) in the SCZ-nct group, with no significant difference between groups (χ^2^ = 1.556; *p* = 0.212). The mean age was 28.24 ± 7.90 years in the SCZ-ct group and 29.58 ± 8.32 years in the SCZ-nct group, with no significant difference (t = 1.082, *p* = 0.280). There was no significant difference between the two groups in terms of educational level (χ^2^ = 4.104, *p* = 0.250). In the SCZ-ct group, 77 patients (32.9%) had a family history of mental disorder, while 15 patients (30.0%) in the SCZ-nct group reported a family history, with no significant difference between the groups (χ^2^ = 0.159; *p* = 0.690). The mean duration of mental disorder was 1.5955 ± 3.528 years in the SCZ-ct group and 1.1616 ± 1.322 years in the SCZ-nct group, with no significant difference (t = -0.856; *p* = 0.393). These results indicate that the two groups were well matched on key demographic and clinical variables, providing a suitable cohort for examining the relationship between CT and SCZ symptom severity.

### Differences in CTQ scores and PANSS scores

To investigate the differences in CT and SCZ symptoms between the two groups described above, we next analysed the CTQ and PANSS scores of the patients (Table 2).

**Table 2:** Comparison of CTQ and PANSS scores between SCZ-ct and SCZ-nct.

	SCZ-ct	SCZ-nct	t	*p*
Emotional abuse	9.74 (3.39)	8.02 (1.66)	− 5.33	< 0.001
Physical abuse	7.92 (2.19)	6.44 (1.16)	− 6.80	< 0.001
Sexual abuse	7.72 (2.34)	5.98 (0.80)	− 9.15	< 0.001
Emotional neglect	13.65 (4.51)	9.94 (2.48)	− 8.09	< 0.001
Physical neglect	11.67 (4.57)	7.64 (1.10)	− 11.96	< 0.001
Total CTQ score	50.70 (8.71)	38.02 (3.89)	− 16.01	< 0.001
Positive syndrome	31.54 (4.45)	29.96 (4.39)	− 2.29	0.023
Negative syndrome	26.41 (5.69)	26.90 (6.02)	0.54	0.588
Total PANSS score	86.58 (9.47)	83.06 (7.93)	− 2.45	0.015

All significant differences remained significant after false discovery rate correction for multiple comparisons.

As expected, the SCZ-ct group had significantly higher total CTQ scores and subscale scores across all five dimensions compared to the SCZ-nct group (all *p* < 0.001; all FDR‑adjusted *p* < 0.001). Specifically, the SCZ-ct group had higher scores on emotional abuse, physical abuse, sexual abuse, emotional neglect, and physical neglect, all with significant differences.

In terms of psychiatric symptom severity, the SCZ-ct group exhibited significantly higher positive syndrome subscale scores on the PANSS compared to the SCZ-nct group (*p* = 0.023; FDR‑adjusted *p* = 0.036). The total PANSS score was also significantly higher in the SCZ-ct group (*p* = 0.015; FDR‑adjusted *p* = 0.030). However, no significant intergroup difference was found in the negative syndrome subscale scores (*p* = 0.588; FDR‑adjusted *p* = 0.588).

These results indicate that patients in the SCZ-ct group, as expected, had higher levels of childhood trauma exposure and more severe positive symptoms than those in the SCZ-nct group.

### Associations between CT and psychiatric symptom severity

#### Total CTQ score and psychiatric symptom severity

A simple linear regression was applied to examine the association between the total CTQ score and total PANSS score. The analysis revealed that the total CTQ score was positively associated with the total PANSS scores (β = 0.169, SE = 0.058, t = 2.919, *p* = 0.004), indicating that higher childhood trauma exposure was associated with more severe psychiatric symptoms. The model accounted for 2.9% of the variance in psychiatric symptom severity (R^2^ = 0.029, adjusted R^2^ = 0.026) and was significant overall (F = 8.518, *p* = 0.004).

#### CTQ subscale dimensions and psychiatric symptom severity

To identify which specific CTQ dimensions were associated with symptom severity, univariate regression analyses were conducted for each CTQ subscale. Only emotional abuse was significantly associated with total PANSS scores after FDR correction (adjusted *p* = 0.033). The other four CTQ subscales did not show significant associations after correction.

Hierarchical regression models were constructed. Model 2 was a two‑block hierarchical regression including only CTQ subdimensions. In Block 1, emotional abuse was positively associated with total PANSS scores (β = 0.400, SE = 0.170, t = 2.351, *p* = 0.019; model fit: R^2^ = 0.019, adjusted R^2^ = 0.016, F = 5.528, *p* = 0.019). In Block 2, after adding the remaining four trauma subscales, emotional abuse remained significantly associated (β = 0.368, SE = 0.173, t = 2.126, *p* = 0.034). The other four CTQ subscales did not show significant independent associations. The full Block 2 model accounted for 5.0% of the variance (R^2^ = 0.050, adjusted R^2^ = 0.033, F = 2.922, *p* = 0.014).

Model 3 was a fully adjusted three‑block hierarchical regression. Block 1 and Block 2 remained identical to Model 2, while Block 3 further incorporated all demographic and clinical covariates. After adjustment, emotional abuse remained significantly associated with total PANSS scores (β = 0.412, SE = 0.177, t = 2.334, *p* = 0.020). None of the other CTQ subscales reached significance. The final model was significant (F = 2.009, *p* = 0.024) and explained 8.2% of the variance (R^2^ = 0.082, adjusted R^2^ = 0.041). Full results are presented in Table 3.

**Table 3:** Linear regression analyses of childhood trauma dimensions and total PANSS score.

Variables	Model 1				Model 2 (Step 1)				Model 2 (Step 2)				Model 3			
	β	SE	t	*p*	β	SE	t	*p*	β	SE	t	*p*	β	SE	t	*p*
Total CTQ score	0.169	0.058	2.919	0.004	–	–	–	–	–	–	–	–	–	–	–	–
Emotional abuse	–	–	–	–	0.400	0.170	2.351	0.019	0.368	0.173	2.126	0.034	0.412	0.177	2.334	0.020
Physical abuse	–	–	–	–	–	–	–	–	0.380	0.278	1.367	0.173	0.394	0.280	1.406	0.161
Sexual abuse	–	–	–	–	–	–	–	–	0.382	0.250	1.528	0.128	0.347	0.252	1.373	0.171
Emotional neglect	–	–	–	–	–	–	–	–	− 0.096	0.134	− 0.716	0.475	− 0.086	0.136	− 0.636	0.525
Physical neglect	–	–	–	–	–	–	–	–	0.243	0.133	1.821	0.070	0.258	0.135	1.907	0.058
Model Fit indices																
R^2^	0.029				0.019				0.050				0.082			
Adjusted R^2^	0.026				0.016				0.033				0.041			
Model F-value	8.518 (*p* = 0.004)				5.528 (*p* = 0.019)				2.922 (*p* = 0.014)				2.009 (*p* = 0.024)			

β = unstandardized regression coefficient; SE = standard error. Model 1: simple univariate regression with total CTQ score as predictor. Model 2: two-block hierarchical regression (Block 1 = emotional abuse; Block 2 added physical abuse, sexual abuse, emotional neglect, physical neglect). Model 3: fully adjusted three-block hierarchical regression (Block 1 and Block 2 identical to Model 2; Block 3 added age, gender, educational level, family history of mental disorder, duration of mental disorder). All variance inflation factors were < 1.25. Significant p-values (*p* < 0.05) are shown in bold.

This finding was further supported via a supplementary linear mixed-effects sensitivity analysis, where rater identity was specified as a random intercept to quantify variability originating from assessor-specific characteristics. This variance decomposition approach differs from formal inter-rater reliability testing, which requires multiple raters to independently evaluate identical participants—a design not implemented in the present study. In this mixed-effects model, emotional abuse remained the sole statistically significant fixed predictor (β = 0.412, *p* = 0.020). The intraclass correlation coefficient reflecting rater-attributable variance was negligible (ICC < 0.001), suggesting minimal measurement variability introduced by systematic differences across raters (Supplementary Table S1). A likelihood ratio test comparing this mixed-effects model with Model 3 showed no significant difference in model fit (χ^2^ = 1.895, *p* = 0.388), further indicating that rater-specific variation exerted little observable impact on the study’s primary estimates (Supplementary Table S2).

In summary, emotional abuse was consistently associated with higher total PANSS scores across all models. The other CTQ dimensions did not show independent associations, and their addition did not significantly improve model fit, suggesting that the shared variance among trauma types warrants cautious interpretation. This association persisted after controlling for rater variability in a linear mixed‑effects model, further supporting the robustness of the finding.

## Discussion

To the best of our knowledge, this is the first study to elucidate the relationship between childhood trauma dimensions and psychiatric symptom severity among drug‑naïve first‑episode patients with schizophrenia. Unlike prior work conducted in samples confounded by illness chronicity and medication exposure, the present design circumvents these potential confounds. This study reached two principal conclusions. Initially, patients with a history of childhood trauma exhibited significantly higher overall symptom severity and positive symptom severity compared to those without such a history, while no significant difference was found in negative symptom severity between the two groups. Based on these observations, this study further explored the potential associations between childhood trauma and symptom severity, revealing that total childhood trauma scores were associated with greater overall symptom severity, and this association was primarily explained by emotional abuse, which emerged as the only trauma dimension maintaining an independent association with symptom severity. Collectively, these findings contribute to a more nuanced understanding of how early adversity shapes symptom expression in schizophrenia and underscore the importance of targeted early intervention efforts that address childhood trauma, particularly emotional abuse.

The first key finding was patients with childhood trauma exhibited greater overall symptom severity, with a preferential association with positive symptoms and no significant difference in negative symptoms, is consistent with a growing body of evidence documenting stronger and more consistent links between childhood trauma and positive symptom dimensions than with negative symptoms^14,17^. This pattern is particularly pronounced in people with first-episode psychosis^11^. These converging findings support the view that childhood trauma may preferentially impact neurodevelopmental pathways implicated in positive symptom generation. According to the neurodevelopmental model of schizophrenia, childhood trauma has been associated with abnormalities in white matter integrity of the superior and inferior longitudinal fasciculi, inferior fronto-occipital fasciculus and occipital part of the corpus callosum radiations, and these abnormalities may disrupt the functions of information integration and salience processing^18^. These neurobiological alterations may contribute to dopaminergic dysregulation and abnormal sensory processing, which in turn may be related to the emergence of positive symptoms such as hallucinations and delusions. From a clinical perspective, these findings imply that a history of CT may help identify patients at increased risk for more severe positive symptom presentations, thereby informing symptom monitoring and treatment planning^19,20^.

A more specific finding was that emotional abuse emerged as the only trauma dimension independently associated with symptom severity. This finding is consistent with recent meta‑analysis identifying emotional abuse as the adversity subtype most strongly associated with psychosis^7^. Unlike physical abuse or sexual abuse, which often involve acute and overt harm, emotional abuse is characterized by chronic emotional deprivation, persistent denial of emotional needs, and devaluation of the individual’s self‑worth within intimate caregiver relationships—experiences that may be particularly detrimental during critical periods of neurodevelopment. Such long‑term emotional maltreatment may undermine the formation of secure attachment and healthy self‑perception in childhood, potentially contributing to enduring deficits in emotional regulation, interpersonal trust and cognitive processing in adulthood. These deficits, in turn, may interact with neurodevelopmental vulnerabilities to contribute to symptom severity^21,22^. At the same time, the addition of the other CTQ dimensions did not significantly improve model fit, and the association may partly reflect shared variance with other forms of childhood adversity. This pattern is consistent with the well‑documented co‑occurrence of multiple trauma types in clinical populations, where different forms of childhood adversity rarely occur in isolation^23,24^. We therefore interpret the finding cautiously: emotional abuse emerged as the dimension most robustly associated with symptom severity, suggesting that it may serve as a particularly informative indicator of an emotionally adverse early environment and a useful focus of clinical assessment in patients with schizophrenia.

Notably, the association between emotional abuse and symptom severity persisted after controlling for demographic and clinical covariates in Model 3. This indicates that the relationship is not simply attributable to measured sociodemographic or illness‑related confounders. However, the proportion of variance explained across all models was modest. Model 1, which included only the total CTQ score, accounted for 2.9% of the variance in total PANSS scores (R^2^ = 0.029). Model 2, which added the other CTQ subscales, accounted for 5.0% (R^2^ = 0.050). Even after incorporating demographic and clinical covariates in the fully adjusted Model 3, the total variance explained reached only 8.2% (R^2^ = 0.082). These findings underscore that childhood trauma is only one of many contributors to symptom heterogeneity in schizophrenia, and genetic predisposition, neurobiological factors, and other psychosocial variables likely account for substantially larger portions of the variance^5,25^.

Taken together, this study indicates that childhood trauma, particularly emotional abuse, is selectively associated with the severity of psychiatric symptoms in drug‑naïve first‑episode patients with schizophrenia. These findings carry translational implications at both the clinical and public health levels. At the clinical level, previous research and current clinical practice guidelines have emphasized the incorporation of psychological assessment as an integral component of routine psychiatric evaluation, and the assessment of childhood trauma constitutes a core element of this process^26–29^. The present findings further corroborate the importance of this direction and support routinely incorporating validated childhood trauma screening instruments, such as the Childhood Trauma Questionnaire, which has been extensively used in psychotic populations^14,16^. Systematic screening may help identify patients with a history of childhood trauma and guide individualized treatment planning. For patients with a documented history of childhood trauma, trauma‑focused cognitive behavioral therapy and integrative trauma‑informed care approaches may complement standard pharmacotherapy by addressing the psychological consequences of early adversity, thereby reducing symptom severity and improving functional outcomes^30–33^. At the public health level, reducing population‑level exposure to childhood trauma is essential to lessening the long‑term burden of severe mental disorders. Evidence‑based approaches that target modifiable risk factors for childhood trauma, including parent training programs, home visitation programs and trauma‑informed care practices integrated within pediatric healthcare networks, have demonstrated effectiveness across diverse healthcare contexts and offer scalable pathways toward this goal^34–36^. Future public health initiatives should prioritize the integration of these strategies across healthcare, education and social service sectors, particularly in settings with elevated risk for childhood trauma.

### Limitations

First, the cross-sectional design limits the ability to draw definitive conclusions regarding the temporal relationship between CT and psychiatric symptom severity. While associations between CT and symptoms were observed, future longitudinal studies may help further elucidate the directionality of these relationships and the developmental trajectories involved.

Second, Childhood trauma was assessed using the retrospective self‑report CTQ. Given that current mental state may influence retrospective accounts, the CTQ was administered only after patients had completed at least 2 weeks of inpatient treatment and were clinically stable. This precaution reduced the likelihood that acute psychotic symptoms shaped responses, although residual symptoms and recall bias may still have had some influence. Future studies would benefit from multi‑method approaches to cross‑validate retrospective self‑reports.

Third, the sample distribution was unbalanced, with the SCZ‑ct group (*n* = 234) larger than the SCZ‑nct group (*n* = 50). This reflects the naturalistic composition of consecutive admissions and the high prevalence of childhood trauma in clinical sample. Power analyses and bootstrap sensitivity analysis confirmed that the core findings were robust to this imbalance. However, it may limit the generalizability of between‑group comparisons. Future studies with more balanced group sizes would be valuable to extend these findings.

Fourth, all participants were recruited from a single psychiatric hospital. While this ensured consistency in diagnostic and assessment procedures, the findings may not fully generalize to other settings. Future multi‑center studies would be valuable to confirm the generalizability of these findings.

Fifth, formal inter-rater reliability testing could not be performed in this study, as no identical participants were independently rated by multiple assessors. The supplementary linear mixed-effects sensitivity analysis only quantified variance attributable to rater differences, rather than measuring overall scoring consistency across raters. Although the rater-level ICC derived from this model was below 0.001 and implied low rater-related variability, we refrain from drawing definitive conclusions about inter-rater scoring agreement and avoid framing this variance decomposition result as a standard inter-rater reliability metric.

## Conclusion

In this sample of drug‑naïve first‑episode patients with schizophrenia, those with a history of childhood trauma exhibited greater overall and positive symptom severity than those without, whereas negative symptoms did not differ. Emotional abuse was the CTQ dimension most consistently associated with symptom severity, and this association persisted after adjustment for other trauma dimensions, demographic and clinical covariates, and rater effects. These findings support the potential value of addressing childhood trauma, particularly emotional abuse, in both clinical practice and public health strategies.

## Supplementary Information

Below is the link to the electronic supplementary material.

Supplementary Material 1

## Data Availability

The datasets generated or analysed during the study are available from the corresponding author upon reasonable request.

## Abbreviations

SCZ: Schizophrenia
CT: Childhood trauma
PANSS: Positive and negative syndrome scale
CTQ: Childhood trauma questionnaire
FDR: False discovery rate
ICC: Intraclass correlation coefficient
SD: Standard deviation
β: Unstandardized regression coefficient
SE: Standard error
R^2^: Coefficient of determination
