# Supplementary material for: Childhood trauma, specifically emotional abuse, is associated with psychiatric symptom severity in drug‑naïve first‑episode patients with schizophrenia
Source: Sci Rep. 2026 Jul 23;16:24736. doi: 10.1038/s41598-026-63324-2 (PMC13458051; doi:10.1038/s41598-026-63324-2)
Supplement: Supplementary file 1 [file 41598_2026_63324_MOESM1_ESM.docx]

**Childhood trauma, specifically emotional abuse, is associated with psychiatric symptom severity in drug‑naïve first‑episode patients with schizophrenia**

Shilong Jia^1^*, Hailuo Liu^2^*, Renyun Zhang^1^, Jie Liu^1^, Sufang Qi^1^, Mengmeng Sun^1^, Limin Yang^1^

**Corresponding Author**

Limin Yang, Department of Psychiatry, Shandong Mental Health Center, Shandong University, Jinan, Shandong, China; Email: yanglm1106@126.com.

**Supplementary Table S1 Linear mixed‑effects model examining the association between childhood trauma dimensions and total PANSS score with rater as a random intercept**

| **Variable** | **Estimate** | **SE** | ***df*** | ***t*** | ***p*** |
| --- | --- | --- | --- | --- | --- |
| (Intercept) | 76.747 | 5.277 | 271 | 14.545 | <0.001 |
| Gender (female) | −0.576 | 1.182 | 271 | −0.488 | 0.626 |
| Age | −0.111 | 0.070 | 271 | −1.599 | 0.111 |
| Duration of mental disorder | −0.138 | 0.183 | 271 | −0.753 | 0.452 |
| Educational level (Primary school) | 1.585 | 3.195 | 271 | 0.496 | 0.620 |
| Educational level (Junior high school) | 2.565 | 3.113 | 271 | 0.824 | 0.411 |
| Educational level (College or above) | −0.828 | 3.304 | 271 | −0.251 | 0.802 |
| Family history of mental disorder (positive) | 0.157 | 1.204 | 271 | 0.130 | 0.896 |
| Emotional abuse | 0.412 | 0.177 | 271 | 2.334 | 0.020 |
| Physical abuse | 0.394 | 0.280 | 271 | 1.406 | 0.161 |
| Sexual abuse | 0.347 | 0.252 | 271 | 1.373 | 0.171 |
| Emotional neglect | −0.086 | 0.136 | 271 | −0.636 | 0.525 |
| Physical neglect | 0.258 | 0.135 | 271 | 1.907 | 0.058 |

Notes: The model included total PANSS score as the dependent variable. All five CTQ subscales (emotional abuse, physical abuse, sexual abuse, emotional neglect, and physical neglect) and demographic/ clinical covariates (age, gender, duration of mental disorder, educational level and family history of mental disorder) were entered as fixed effects. Rater was included as a random intercept to account for potential variability attributable to individual assessors. Education level was dummy‑coded with senior high school as the reference category. Gender was coded as 1 = male, 0 = female. Family history of mental disorder was coded as 1 = positive, 0 = negative.

**Supplementary Table S2 Model fit indices and likelihood ratio test for linear mixed‑effects model and model 3**

| **Variable** | **AIC** | **BIC** | **-2*log(L)** | **Chisq** | ***p*** |
| --- | --- | --- | --- | --- | --- |
| Linear mixed‑effects model | 2077.3 | 2132.0 | 2047.3 |  |  |
| Model 3 | 2079.4 | 2141.4 | 2045.4 | 1.895 | 0.388 |

Notes: AIC：Akaike Information Criterion; BIC：Bayesian Information Criterion; -2*log(L)：−2 × Log-Likelihood.
